# Smartphone Versus Pen-and-Paper Data Collection of Infant Feeding Practices in Rural China

**DOI:** 10.2196/jmir.2183

**Published:** 2012-09-18

**Authors:** Shuyi Zhang, Qiong Wu, Michelle HMMT van Velthoven, Li Chen, Josip Car, Igor Rudan, Yanfeng Zhang, Ye Li, Robert W Scherpbier

**Affiliations:** ^1^Department of Integrated Early Childhood DevelopmentCapital Institute of PediatricsBeijingChina; ^2^Global eHealth UnitDepartment of Primary Care and Public HealthImperial College LondonLondonUnited Kingdom; ^3^Centre for Population Health Sciences and Global Health AcademyUniversity of Edinburgh Medical SchoolEdinburghUnited Kingdom; ^4^Section of Health and Nutrition, Water, Environment and SanitationUNICEF ChinaBeijingChina

**Keywords:** Data collection, health survey, questionnaires, infant feeding, smartphone

## Abstract

**Background:**

Maternal, Newborn, and Child Health (MNCH) household survey data are collected mainly with pen-and-paper. Smartphone data collection may have advantages over pen-and-paper, but little evidence exists on how they compare.

**Objective:**

To compare smartphone data collection versus the use of pen-and-paper for infant feeding practices of the MNCH household survey. We compared the two data collection methods for differences in data quality (data recording, data entry, open-ended answers, and interrater reliability), time consumption, costs, interviewers’ perceptions, and problems encountered.

**Methods:**

We recruited mothers of infants aged 0 to 23 months in four village clinics in Zhaozhou Township, Zhao County, Hebei Province, China. We randomly assigned mothers to a smartphone or a pen-and-paper questionnaire group. A pair of interviewers simultaneously questioned mothers on infant feeding practices, each using the same method (either smartphone or pen-and-paper).

**Results:**

We enrolled 120 mothers, and all completed the study. Data recording errors were prevented in the smartphone questionnaire. In the 120 pen-and-paper questionnaires (60 mothers), we found 192 data recording errors in 55 questionnaires. There was no significant difference in recording variation between the groups for the questionnaire pairs (*P *= .32) or variables (*P *= .45). The smartphone questionnaires were automatically uploaded and no data entry errors occurred. We found that even after double data entry of the pen-and-paper questionnaires, 65.0% (78/120) of the questionnaires did not match and needed to be checked. The mean duration of an interview was 10.22 (SD 2.17) minutes for the smartphone method and 10.83 (SD 2.94) minutes for the pen-and-paper method, which was not significantly different between the methods (*P *= .19). The mean costs per questionnaire were higher for the smartphone questionnaire (¥143, equal to US $23 at the exchange rate on April 24, 2012) than for the pen-and-paper questionnaire (¥83, equal to US $13). The smartphone method was acceptable to interviewers, and after a pilot test we encountered only minor problems (eg, the system halted for a few seconds or it shut off), which did not result in data loss.

**Conclusions:**

This is the first study showing that smartphones can be successfully used for household data collection on infant feeding in rural China. Using smartphones for data collection, compared with pen-and-paper, eliminated data recording and entry errors, had similar interrater reliability, and took an equal amount of time per interview. While the costs for the smartphone method were higher than the pen-and-paper method in our small-scale survey, the costs for both methods would be similar for a large-scale survey. Smartphone data collection should be further evaluated for other surveys and on a larger scale to deliver maximum benefits in China and elsewhere.

## Introduction

Undernutrition in infants and young children is highly prevalent in low- and middle-income countries and results in substantial mortality and morbidity [1]. Inadequate breastfeeding and complementary feeding are the key factors causing undernutrition in infants, which ultimately affects child survival [2]. Globally, suboptimal breastfeeding is estimated to be responsible for 1.4 million child deaths and 44 million disability-adjusted life-years [1]. Therefore, improving infant feeding practices in children from 0 to 23 months of age is critical to improve nutrition, health, and development of the children.

Breastfeeding and complementary feeding practices are key indicators for child health [3]. Accurate data on feeding practices is extremely important to start appropriate health interventions that can improve children’s health. The Maternal, Newborn, and Child Health (MNCH) household survey (unpublished data, 2009) is an instrument for collecting data on the coverage of key child health interventions, delivery channels, reasons for coverage failure, and health expenditures. Originally, this instrument was developed by the World Health Organization (WHO) as a paper-based questionnaire for resource-limited settings and had been used in Cambodia, Papua New Guinea, and Vietnam [4]. One of the MNCH household survey modules is on breastfeeding and nutrition aiming to collect feeding information of children aged 0 to 23 months. In 2009, WHO and UNICEF jointly developed the guidelines on indicators for assessing infant and young child feeding practices [3], setting up a series of international standard infant and young child feeding coverage indicators.

For decades, pen-and-paper-based data collection has been the standard method for household surveys. However, this has several problems, including data collection and entry errors and the high costs for storage and double entry of data [5]. In the past 20 years, electronic methods of data collection have been developed on handheld devices such as personal digital assistants (PDAs) and more recently on mobile phones. Worldwide there are now about 6 billion mobile phones, of which 4.5 billion can be found in developing countries [6]. The growth in mobile phone subscriptions is led by China and India, which now have over 30% of the world’s subscribers [7]. The use of mobile devices for the delivery of health care, also known as mHealth [8,9], has increasingly gained attention over the past years. However, a limited number of studies have evaluated the use of mobile phones as a data collection tool in developing settings. Our literature searches in electronic English-language health databases (The Cochrane Central Register of Controlled Trials, PubMed, EMBASE, WHO Global Health Library regional index, PsycINFO, Web of Science, MobileActive, KIT Information Portal, and mHealth in Low-Resource Settings) found 9 studies using mobile phones for health data collection [10-17], and we found no studies in the Chinese literature (Wanfang Data and the China National Knowledge Infrastructure).

Smartphones may be more suitable than low-end mobile phones for data collection, as smartphones have larger screens and can more easily accommodate complex functions (such as wireless uploading and downloading, screen touch typing, and photo or video capturing). Rapid developments in technology and falling prices of handsets make smartphones more accessible for data collection in developing settings. Smartphones share the advantages PDAs have for data collection, for example, the ability to combine the processes of data recording and data entry [18]. Smartphone software can be programmed to skip questions and give alerts when a question is answered incorrectly, which further improves data accuracy. Smartphone data collection may provide better data quality, less time consumption, and lower costs than with pen-and-paper data collection. However, the use of smartphones has some drawbacks, including that data can become corrupted when the device is damaged, and replacement costs are relatively high when the device is lost or damaged. Moreover, most surveys were originally designed for pen-and-paper use (the reference standard), and validation of questionnaires is required. There could be response bias between paper and electronic questionnaire versions [19], as patients may respond differently to questionnaires in different formats [20].

It is unknown whether smartphones can be effectively used for household survey data collection in rural China. This study aimed to compare the use of smartphones with the use of pen-and-paper for data collection of infant feeding practices with the MNCH household survey. We evaluated differences in data quality (data recording, data entry, open-ended answers, and interrater reliability), time consumption, costs, interviewer’s perception, and problems encountered.

## Methods

### Study Design

This study compared two methods for MNCH household survey data collection: smartphone versus pen-and-paper. We randomly assigned mothers of infants aged 0 to 23 months to a smartphone or a pen-and-paper group. A pair of interviewers simultaneously interviewed mothers on infant feeding practices. Both interviewers used the same method, either the smartphone or the pen-and-paper method. The interviewers each recorded the mothers’ responses separately at the same time. One interviewer asked the questions and the other interviewer assisted in providing details. We instructed the interviewers to change the leading and assisting roles in every interview. We compared the two data collection methods for differences in data quality (data recording, data entry, open-ended answers, and interrater reliability), time consumption, costs, perception of interviewers, and problems encountered. We undertook a small 2-day pilot study in July 2011 to test this setup.

### Study Setting

We carried out the surveys in four village clinics in Zhaozhou Township, Zhao County, Hebei Province, China. More detailed information on the study setting can be found in Multimedia Appendix 1.

### Participants

There were two types of participants: (1) interviewers, who interviewed the mothers in pairs, and (2) mothers, who were interviewed on their infant feeding practices. We recruited 10 students from Hebei Union University School of Public Health (1 second-year and 9 third-year students) as interviewers. We visited village doctors (in China, village doctors are familiar with all births in their catchment area and monthly report all pregnant women and newborns to their township hospital) and asked them to identify (by review of their records) all eligible mothers on their list and to invite them to come to the clinic. Mothers were eligible if they had children less than 24 months old; we excluded caregivers who were not the mother of the child. If there were more than 2 children of eligible age in a family, we collected information on the youngest child. Eligible mothers were requested to ask their neighbors to visit the clinic as well. The team visited village clinics one by one until we reached our sample size of 120.

### Training of Interviewers

Our interviewers had experience with the MNCH household survey; 3 of them had participated in our pilot smartphone questionnaire study in July 2011 and all 10 participated in a baseline MNCH household survey (with a sample size of 1600) in August 2011. Although the interviewers were familiar with the survey, we provided them with additional training to reinforce their skills. The supervisors (study team members) thoroughly trained the interviewers on the use of the smartphone and pen-and-paper methods for 2 days. The training course included communication skills, explanation of questionnaires, demonstration, role plays, practice interviews with mothers, and group discussion throughout the course. Interviewers were encouraged to ask questions when they experienced any problem.

### Recruitment

The study took place over 2 days in September 2011; on the first day we recruited mothers in three village clinics, and on the second day we reached our sample size in the fourth village. When a mother arrived in a village clinic, the village doctor informed her about the study and referred mothers who were interested in study participation to our supervisors. The supervisors obtained oral informed consent from the mother before the study. The interviewers obtained written informed consent and then questioned the mother in one of the five separate village clinic rooms. We gave each mother a towel (worth ¥5, equal to US $0.79 at an exchange rate of 6.3 on April 24, 2012) for her participation.

### Randomization and Allocation

A supervisor gave mothers an identification number (the first mother was given number 1, the second mother number 2, and so on). Prior to the study we used Excel (version 2007; Microsoft Corporation, Redmond, WA, USA) to randomly assign each number to either the smartphone or the pen-and-paper group. Each of the 10 interviewers randomly drew a lot and was assigned to 1 of the 5 interview pairs in which they worked during the study. The 5 interview work pairs took turns when interviewing the mothers.

### Data Collection and Entry Process

#### Pen-and-Paper Method

For mothers who were randomly assigned to the pen-and-paper method, an assigned pair of interviewers asked questions that were printed on a paper questionnaire. Each of the 2 interviewers separately recorded the interviewee’s response with a pen on a copy of the questionnaire. A supervisor collected the completed paper questionnaires and checked them immediately after the interview to ensure that any missing information or errors could be corrected before the mother left. Two students, who had experience with data entry, separately entered data with EpiData 3.1 (EpiData Software, Odense, Denmark). We compared the two files and resolved discrepancies by checking the original questionnaires.

#### Smartphone Method

**Figure 1:**
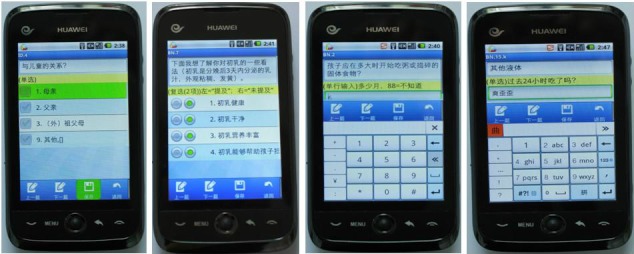
Screen shots of single-choice, multichoice, number-filling, and text-filling questions for the smartphone questionnaire.

For mothers who were randomly assigned to the smartphone method, an assigned pair of interviewers questioned the mothers by following the instructions on the smartphone. We used a Chinese smartphone (C8600; Huawei, Shenzhen, China) with an Android 2.2 system. Each interviewer recorded the mother’s response by touching the smartphone screen. The smartphone program checked the questions automatically in real time. The smartphone program showed a warning when an answer was out of range. Three questions could be automatically skipped based on the response of previous questions. Most of the questions were multiple-choice questions with one-response or multiple-response answers. Some questions required a number to be filled in, and for other variables one of the answer options of multiple-response answers was open ended (which made it possible to type in Chinese characters for the smartphone questionnaires or write down characters for the pen-and-paper questionnaires). Figure 1 shows screen shots of the main four types of questions. More detailed information on the smartphone questionnaire can be found in Multimedia Appendix 1.

### Survey

We used the Breastfeeding and Nutrition module of MNCH household survey. This is a WHO standard household survey, which we translated into Chinese. We have used it over the past 2 years in several studies in Zhao County and made minor changes to make it appropriate for the Chinese context. More detailed information on the survey can be found in Multimedia Appendix 1.

### Outcomes

Our primary outcome was data quality, which we defined as data recording errors and data entry errors in the pen-and-paper questionnaires; interrater reliability; and open-ended answer differences between the smartphone and pen-and-paper methods. Secondary outcomes were the differences between the two methods in time consumption for the data recording and entry, costs, interviewers’ perceptions, and problems they encountered.

#### Data Quality

##### Data Recording Errors of the Paper Questionnaires

We report on data recording errors for the paper questionnaire only, as the smartphone program automatically alerted the interviewer when a mistake occurred (and therefore there were zero data recording errors in the smartphone questionnaires). The supervisors checked and corrected missing values or errors at the end of the interview. After the fieldwork, 1 of the supervisors filled in an error summary form with the frequency of all types of errors and numbers of questionnaires with errors. We counted the errors in Excel 2007 with the COUNT function.

##### Interrater Reliability

We defined interrater reliability as the percentage of overlap in recording between the 2 interviewers who interviewed the same mother using the same method [21].

##### Data Entry Errors of Paper Questionnaires

We report data entry errors for the pen-and-paper questionnaires only, as the smartphone questionnaire responses were automatically transferred to the database after uploading (according to the information technology experts, the smartphone data were not changed during transfer into the database). For the pen-and-paper questionnaire, though 2 people entered and checked the data separately, errors could still occur.

##### Open-Ended Answers

We counted the number of Chinese symbols for the 24 questions that had one open-ended answer.

#### Time Consumption for Data Recording and Entry

For the pen-and-paper questionnaire, the interviewers recorded the starting time and ending time of the interview. For the smartphone questionnaire, the software automatically recorded the duration of the interview. In addition, we recorded the time consumption of data entry and data cleaning for the pen-and-paper group.

#### Costs

To assess and compare costs, we used the same cost categories for the pen-and-paper and smartphone questionnaires categories (eg, training, travel, and accommodation). For the pen-and-paper method, we estimated the costs of printing and transporting the questionnaire, stationery, and data entry. In our smartphone costs assessment, we used an estimated local market price for renting the smartphone and the software. To compare the pen-and-paper and smartphone methods, we calculated cost by individual questionnaire. In addition, we used the cost data of two large-scale household surveys (sample sizes of 1200 and 1600) to show the costs for larger-scale surveys.

#### Perceptions of the Interviewers

Before the fieldwork, we gave each interviewer pen-and-paper questionnaires. We asked them to fill in the questionnaire and to give it back to the supervisor after the fieldwork. The questionnaire included general information (eg, age, sex, and education), experience with using a mobile phone or a smartphone, and perceptions of using the two methods for the interviews. We asked them if they liked the smartphone and the pen-and-paper methods, and the interviewer could respond on a scale from 1 to 5 (1, very bad; 2, bad; 3, ok; 4, good; and 5, very good). After the fieldwork, our supervisors and all the interviewers had a 30-minute group discussion about the differences between the pen-and-paper survey and the smartphone survey (such as time needed for an interview, which method was easier to communicate with the interviewees, and responses of the mothers during the interview).

#### Problems Encountered

We instructed the interviewers to record problems, such as loss of pens and program errors, as soon as possible on standard forms during the fieldwork. The supervisors collected all the forms after the fieldwork and wrote down the problems.

### Analysis

#### Quantitative Analysis

We carried out statistical analysis with SPSS 16.0 for Windows (IBM Corporation, Somers, NY, USA). For data recording errors, we manually counted all the errors from the error summary form, which was filled in by our supervisors in the field. For interrater reliability, we manually compared all completed pen-and-paper questionnaires and smartphone questionnaires within each pair and report the percentage variation. We calculated the difference in interrater reliability between two groups with the chi-square test and we report percentages. We did not analyze interrater reliability with kappa statistics, as we were unable to use a test–retest design for this study. For data entry errors we compared the two files using EpiData 3.1, which gave a report on the nonmatching questionnaires. We report the differences in percentages. For time consumption, we compared the average time duration of data collection in a work pair for the two groups by the independent-samples *t *test. We tested whether the time consumption was normally distributed. We report the mean difference and standard deviation, and we used a significance level of .05. For costs, we compared the total costs between the two methods. For interviewers’ perceptions of the smartphone and pen-and-paper questionnaires, we calculated the median of the scale from 1 to 5.

#### Qualitative Analysis

For interviewers’ perceptions, we analyzed the transcription of the recorded discussion and noted all identified issues. We list all the problems that were experienced by the interviewers.

### Sample Size

We based our sample size on the data entry errors. We assumed that the data entry error rate would be 30% for the pen-and-paper method and 0% for the smartphone method. We used an alpha error of .05 and beta error of .20 and calculated that the sample size would be 120 with 60 mothers per method. We assumed that 10% of the mothers would decline to participate or withdraw from the study. Therefore, we planned to recruit 67 mothers per method.

### Ethical Approval

We obtained ethical approval from the Ethical Committee of the Capital Institute of Pediatrics in Beijing. This is a comparison study, which does not assess an intervention, and therefore is not registered with a randomized trial registry.

### Data Security

We encrypted the data stored in the smartphones when the data were uploaded. The data could be decrypted only with special software. When the interviewer completed the questionnaire, the data were wirelessly uploaded into an Excel database via the Internet server and then saved in the memory card of the smartphone as a text file (for a backup). Only the supervisors could enter the database and make the necessary changes before the data were uploaded. After the data were uploaded, no changes could be made to the database. The supervisors collected the smartphones at the end of each fieldwork day and returned the smartphones (cleared of the data that were entered during the previous day) to the interviewers in the morning. The supervisors collected the completed paper questionnaires when the interview was finished and stored the questionnaires in a safely locked box.

## Results

**Figure 2:**
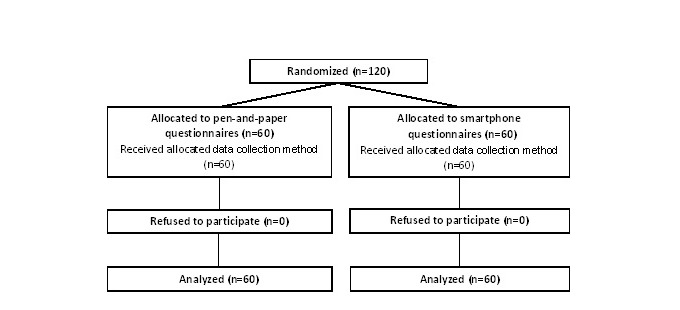
Flow of study participants.

The village doctors informed 120 mothers who visited the village clinics about the study, and all mothers agreed to participate. Figure 2 shows a flow diagram of the recruitment of the 120 mothers: 60 received the paper-and-pen version and 60 received the smartphone version. Table 1 lists the number of interviewed mothers per village. The age and sex ratio of the mother’s youngest infants were similar in the two groups.

**Table 1:** Characteristics of interviewed mothers’ child and number of mothers per village.

Characteristic		Total	Smartphone group	Pen-and-paper group	*P *value
**Children**					
	Age (days), mean (SD)	353.88 (174.65)	323.95 (149.86)	383.82 (192.99)	.06^a^
	Sex (male/female)	61/59	32/28	29/31	.59^b^
**Number of mothers**					
	Total	120	60	60	
	Village 1	25	16	9	
	Village 2	50	23	27	
	Village 3	24	11	13	
	Village 4	21	10	11	

^a ^2-tailed *t *test.

^b ^Pearson chi-square test.

### Data Quality

#### Recording Errors of Pen-and-Paper Questionnaires

Table 2 demonstrates that in 120 copies of the pen-and-paper questionnaires, 55 questionnaires contained errors. The most frequent error was a missing confirmation of the default option, which was found 156 times in 49 questionnaires.

**Table 2:** Recording errors in pen-and-paper questionnaires.

Type of error		Immediately after interview		Before data entry		Total	
		No. of questionnaires with error(s)	No. of errors	No. of questionnaires with error(s)	No. of errors	No. of questionnaires with error(s)	No. of errors	
1	Missing confirmation of default option	49	156	0	0	49	156
2	Missing survey date	1	1	0	0	1	1
3	Wrong response for checking question	2	2	0	0	2	2
4	Two options circled	2	2	0	0	2	2
5	Wrong option chosen	3	4	0	0	3	4
6	Missed question	2	2	1	4	3	6
7	More than one option circled in single-choice question	1	1	0	0	1	1
8	Wrong ID number for interviewer	0	0	7	8	7	8
9	Wrong date	0	0	1	1	1	1
10	Wrong setting for database	0	0	11	11	11	11
Total		51^a^	168	20^a^	24	55^a^	192	

^a ^Total numbers of questionnaires with error(s). A questionnaire could have more than one error, but was counted as one copy. Therefore, the total number of questionnaires does not equal the total number of all types of errors.

#### Interrater Reliability Within Interviewer Pairs

We assessed the interrater reliability for the one-response and multiple-response answer variables and the number variables; this can be found in Table 3. The supervisors checked the two records for each mother and judged whether they were the same. For 35 of the 120 questionnaire pairs (20 pairs in the pen-and-paper group and 15 pairs in the smartphone group), there was no recording variation in the database, which was not significantly different between the groups (*P *= .32). There were 186 variables in the smartphone and 184 in the pen-and-paper questionnaire. In the smartphone questionnaire, 134 of 186 variables (72.0%) did not have any recording variation. In the pen-and-paper questionnaire, 126 of 184 variables (68.5%) did not have any recording variation. This was not significantly different between the groups (*P *= .45).

**Table 3:** Interrater reliability within interviewer pairs.

	Smartphone number/ total number	Pen-and-paper number/ total number	*P *value^a^
Questionnaire pairs with no recording variations	15/60	20/60	.32
Variables with no recording variations	134/186 (72.0%)	126/184 (68.5%)	.45

^a ^Pearson chi-square test.

#### Open-Ended Answers

We manually counted the characters recorded in variables for the open-ended questions. There were 48 characters in the smartphone questionnaires and 76 in the pen-and-paper questionnaires.

#### Data Entry Errors of Pen-and-Paper Questionnaires

EpiData 3.1 showed that 65.0% (78 of 120 questionnaires) of the pen-and-paper records did not match and needed to be checked.

### Time Consumption

**Figure 3:**
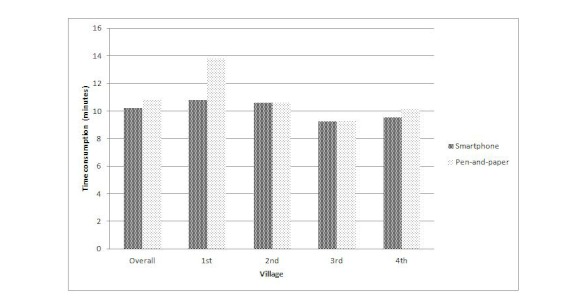
Time consumption for the conducting the interviews overall and per village for the smartphone and pen-and-paper methods.

Figure 3 and Multimedia Appendix 2 (xTable MA3) show that the mean duration of an interview was 10.60 (SD 2.49) minutes, with 10.22 (SD 2.17) minutes for the smartphone method and 10.83 (SD 2.94) minutes for the pen-and-paper method. The mean interview duration was not significantly different between the methods (*P *= .19).

In the first village, the interviewers spent significantly more time for the pen-and-paper method (mean 13.78 minutes, SD 3.70) than for the smartphone method (mean 10.78 minutes, SD 2.37) for an interview (*P *= .02). We found no significant difference between the two methods in the following three villages.

For the pen-and-paper method, database completion took 16 hours (including data entry, checking, and data cleaning). For the smartphone method, database completion took half an hour. This can be found in Table 4.

**Table 4:** Time consumption for the survey process for the two methods.

Phase	Smartphone method	Pen-and-paper method
Preparation	7 days for printing	7 days for programming and installment for a team of 3 information technology engineers
Training	2 days	2 days
Fieldwork	2 days	2 days
Data pooling	3 hours for 2 persons to do double data entry, and 5 hours for 2 persons to check and clear the data	0.5 hour for 1 person

### Costs

Table 5 displays the total costs and costs per questionnaire, which are divided into two parts, logistics and questionnaire work. We estimated the costs based on local market prices. We spent ¥17,200 (US $2730) for the smartphone group (survey 1) and ¥9970 (US $1582) for the pen-and-paper group (survey 2). This included all items for preparation, training, fieldwork and data collection, and logistics. We used the following estimates: leasing the software cost ¥600 (US $95) per week (the software could not be leased per day); renting the sever cost ¥3000 (US $476) per week; labor cost ¥100 (US $16) per hour and ¥300 (US $48) per day; and postage was ¥336 (US $53) per 40 kg parcel (delivered by the National Post Office). Table 5 shows that the costs of both methods were similar for larger-scale household surveys. Multimedia Appendix 2 (xTable MA4) shows the general information on the basis of which we estimated the costs for our two methods (survey 1 and 2) and for two larger-scale surveys (one pen-and-paper and one smartphone).

**Table 5:** Costs (in US $1000) of four surveys in Zhao County, China.

Survey number	Type	No. of interviewees	Costs		Item costs					
					Logistics		Hardware and mailing		Data pooling	
			Total	Per questionnaire	Total	Percentage of all costs (%)	Total	Percentage of all costs (%)	Total	Percentage of all costs (%)
1	Smartphone	60	2.70	22.50	1.27	47.09	1.41	52.33	0.02	0.58
2	Pen-and-paper	60	1.57	13.05	1.27	81.24	0.04	2.71	0.25	16.05
3	Smartphone	1600	41.57	25.98	34.82	83.76	6.59	15.86	0.16	0.38
4	Pen-and-paper	1200	28.52	23.77	25.56	89.60	0.71	2.47	2.26	7.93

### Perceptions of the Interviewers

We analyzed 9 of the 10 questionnaires given to the interviewers, as we received 1 blank questionnaire. Of these 9 interviewers, 8 had experience with doing pen-and-paper surveys before this study, and 4 of the 9 interviewers had experience with using a smartphone. On the question about whether the interviewers liked the survey method, their median score was 4 for the smartphone and 3 for the pen-and-paper method. A total of 4 of the interviewers marked the smartphone 1 point higher than the pen-and-paper, and 5 gave the same mark to the smartphone and the pen-and-paper method.

All the interviewers actively participated in the group discussions. For the pen-and-paper questionnaires, the main issues were being afraid of skipping questions, having to write down a lot on the paper that could waste time, perceiving a high risk of missing options that could not be easily found in the fields, having to carry the heavy questionnaires, and transportation difficulties. The only issue the interviewers identified using the smartphone was that if the program was unstable they could not go forward in the questionnaire. The interviewers experienced the following benefits using the smartphone: the automatic skipping function and error alerts took away the interviewers’ fear of making mistakes, one question per screen put more focus on the communication with the mother during the interview, the smartphone was portable and easy to handle, and data upload was quick. The interviewers mentioned that the mothers said that using the smartphone method was more modern and quicker than using the pen-and-paper method.

### Problems Encountered

In the pen-and-paper group, only one abnormal event was recorded: an interviewer lost her pen and got a new pen from a supervisor immediately. In the smartphone group, five cases of abnormal conditions were recorded. All of them were about the system’s stability, such as that the system halted for a few seconds or that it shut off. We did not find that these recorded abnormalities caused data to be lost.

## Discussion

### Principal Results

Our study showed that using the smartphone to collect data on breastfeeding and nutrition, when compared with the pen-and-paper method, eliminated data recording and entry errors, had similar interrater reliability, and took an equal amount of time per interview. Fewer Chinese characters were entered in the smartphone questionnaire, which may indicate that the smartphone was less suitable for open-ended answers. While the costs for the smartphone method were higher than for the pen-and-paper method in our small-scale survey, the costs for both methods would be similar for a larger-scale survey. The smartphone method was acceptable to interviewers, and after pilot testing we encountered only minor problems.

### Limitations

Our study had some limitations. First, it was hard to validate each interview when comparing the two methods. The test and retest methods are not feasible for the Breastfeeding and Nutrition module, which is based on 24-hour recall information. Therefore, we allocated 2 interviewers using the same method to interview the same mother, and then compared the interrater reliability of the two methods. However, different responses may have been given to the interviewers depending on the survey method (for example, the interviewers may have taken pen-and-paper questionnaires more seriously). We were unable to analyze this. Second, our sample size was relatively small, which may explain the relatively high standard deviations in the time consumption. Also, this may limit the generalizability of our results to other settings. However, at the time of development of the study, we did not have a better indicator than the data entry error assumptions. Third, we do not know how the mothers perceived the interview, as we did not collect data on the perceptions of mothers. However, the interviewers reported that the mothers found the smartphones modern and quicker. Fourth, all our interviewers were medical students and had experience with questionnaire interviewing. All were young and could easily learn how to use the smartphone; this may be different for data collectors in nonstudy situations. We expect that this will be a minor problem, as smartphone ownership is rapidly increasing. Strengths of our study include our pilot test of the smartphone software, which ensured that the system worked, thorough training of our interviewers, and our experience with undertaking the MNCH survey in Zhao County.

### Comparison With Prior Work

While mobile devices have been used for health data collection over the past 20 years, we are unaware of any study using a mobile device or smartphone for data collection in China. In other countries, prior to the rapid development of smartphones, many studies used handheld computers such as PDAs for data collection [22-25]. A review showed that paper- and computer-collected, patient-reported outcomes are equivalent for both methods [26]. Previous studies found that data record and entry errors did not occur when an electronic device was used for data collection [27,28], which we confirmed in our study. A study demonstrated that the use of PDAs reduced data entry and transfer time by 23% [28]. Another study showed that it took less time to complete a questionnaire in the PDA group than in the pen-and-paper group [29]. Our study found that the smartphone can effectively use real-time upload and backup, and can prevent data loss problems, which was similar to the findings of a South African study using mobile phones as a data collection tool [5]. A study of an Android-based mHealth system found that users of the system felt it was easy to use and that it facilitated their work [30], which our interviewers also experienced.

In China, an estimated 70% of the population own a mobile phone, and they are widely used in both urban and rural areas [7]. In Zhao County, approximately 75% of the population and nearly all households have at least one mobile phone. Rapid developments in information and communication technology make Internet access increasingly affordable to people in China. The current third-generation network covers most of the counties (except for some remote areas) and facilitates data upload. The smartphone we used costs ¥1500 (US $230). As prices of handsets are falling rapidly, future studies may be able to use even cheaper smartphones with more functions. Additional functions such as a global positioning system, storing pictures and videos, taking photos, and recording sound could facilitate data collection.

### Conclusion

This study is the first to show that smartphones can be successfully used for data collection in a rural setting in China. Accurate data are essential for the success of any public health survey and to inform appropriate interventions. Smartphone data collection can improve data quality by preventing data recording and entry errors. Also, increased efficiency at the data recording and entry stage is an important benefit of the smartphone method compared with the pen-and-paper method. This could lead to substantial time and cost savings. Smartphone data collection should be further evaluated for other surveys and on a larger scale to deliver maximum benefits to China and elsewhere.

## Multimedia Appendix 1

More detailed information on the Methods section of the paper.

## Multimedia Appendix 2

More detailed information on the Results section of the paper.

## Abbreviations

MNCH: Maternal, Newborn, and Child Health
PDA: personal digital assistant
WHO: World Health Organization
