# Supplementary material for: Smartphone Versus Pen-and-Paper Data Collection of Infant Feeding Practices in Rural China
Source: J Med Internet Res. 2012 Sep 18;14(5):e119. doi: 10.2196/jmir.2183 (PMC3510690; doi:10.2196/jmir.2183)

More detailed information on the Methods section of the paper

Study setting

Hebei province is located in the northern part of China and Zhao County is located in the middle-south part of Hebei Province. The annual per capita net income of rural residents in Hebei province was 5,958 Yuan (946 USD) in 2010 (which is close to the national average of 5,919 Yuan (939 USD)), and 6,464 Yuan (1026 USD) for residents of Zhao County (data from the Zhao County statistics bureau, unpublished). Zhao County has a total population of 571,000, of which 60,688 in Zhaozhou Township (unpublished data). There are four county level hospitals, 16 township hospitals, and 280 village clinics in Zhao County.

Survey

The Breastfeeding and Nutrition module of the MNCH survey included general information questions with 17 variables (answer options) and 54 questions with 208 variables on four feeding topics (breast feeding, complementary feeding, information on infant feeding and hand washing). Table MA1 displays the questionnaire items. Most of the questions were multiple-choice questions with one-response (Figure MA1) or multiple-response answers (Figure MA2). Some questions required a number to be filled in (Figure MA3), and for other variables one answer option of multiple-response answers was open-ended (Figure MA4; this was the option 'if other, please specify', which made it possible to type in Chinese characters for the smartphone questionnaires or write down characters for the pen-and-paper questionnaires). The types and numbers of variables are listed in Table MA2.

Pen-and-paper survey

The pen-and-paper questionnaire had 208 variables. For 167 variables, one answer option had to be chosen for one-response answers (40 variables) and multiple response answers (127 variables). For the other 41 variables, 17 required a number to be filled in and 24 variables had an open-ended response answer

Smartphone survey

The smartphone questionnaire had 210 variables; two more than the pen-and paper questionnaire. We added two variables to the multiple-response answers; these were the ones which were most frequently reported in the open-ended answers during our pilot study. Another difference was that 127 variables required a ‘yes’ or ‘no’ answer and were automatically set on ‘no’ in the smartphone; we will refer to these as ‘default options’ (which can be found in the screenshot on the right of Figure 1 in manuscript). The interviewer could change the variable to ‘yes’ depending on the mother’s response.

Table MA1 Questionnaire items

General information	● Child's name, sex, birth date and age
	● Mother's name
	● Interviewer's name and survey date
1. Breast feeding	● Duration for exclusive breastfeeding
	● Feeding on demand
	● Breastfeeding initiative
	● Colostrum
	● Care seeking for breastfeeding problem
2. Complementary feeding	● Food density
	● Meal amount
	● Responsive feeding
	● Introduction of complementary feeding
	● 24 hour recall on dietary survey
3. Information on infant feeding	● Infant feeding information channels
4. Hand washing	● Hand washing on the day

Table MA2 Types and numbers of variables in the smartphone questionnaire and pen-and-paper questionnaire

Variable type	Smartphone (no.)	Pen-and-paper (no.)
➢ For General information	17	17
➢ To judge skipping conditions	0	3
➢ Breastfeeding and Nutrition	210	208
● One-response answer (Figure 1)	40	40
● Multiple-response answer (Figure 2)	127	127
● Number (Figure 3)	17	17
● Open-ended as ‘if other, please specify’ (Figure 4)	24	24
● Added after pilot	2	0

Figure MA1 Question with one response answer

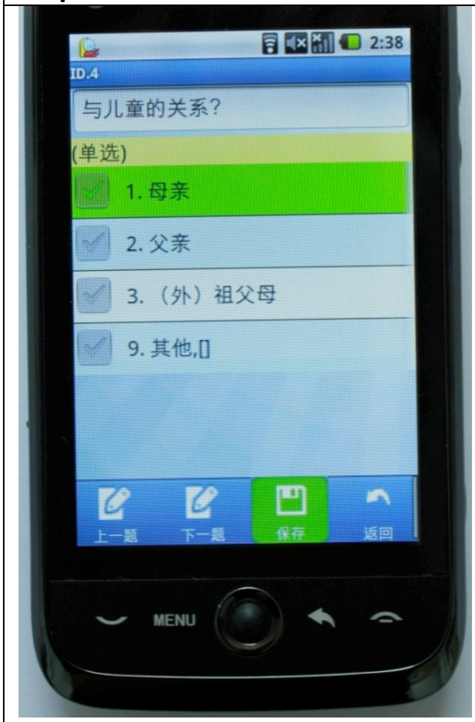

Figure MA2 Question with multiple response answer

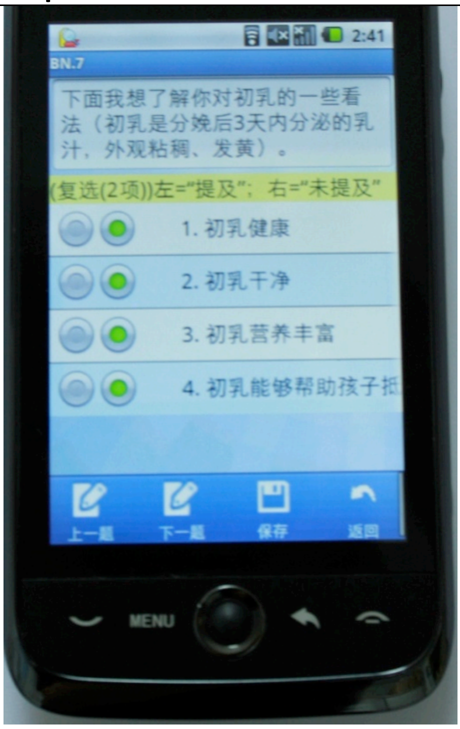

Figure MA3 Question for which the answer needed a number

Figure MA4 Question with one answer option for Chinese

<table>
<tr><td></td><td>character(s)</td></tr>
<tr><td>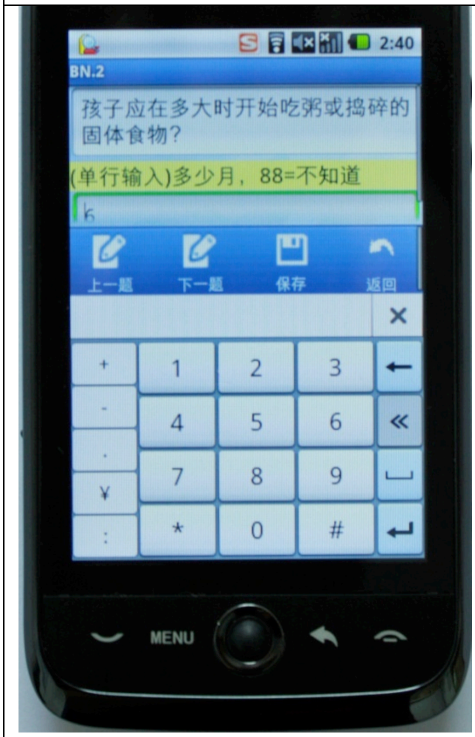
</td><td>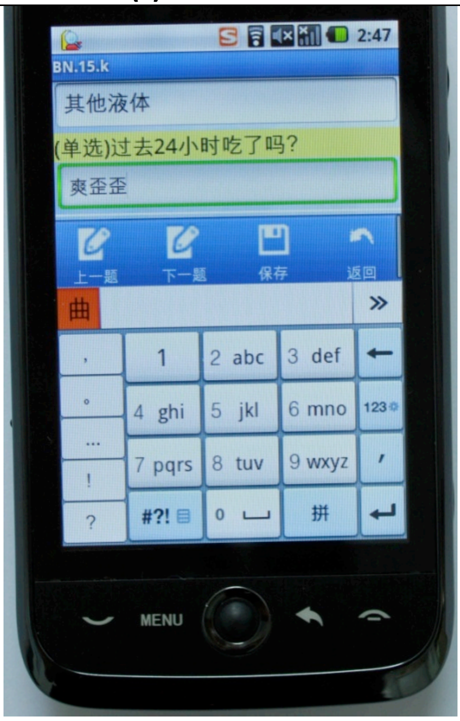
</td></tr>
</table>

Figure MA5 represents a flowchart of the smartphone data process. Together with an information technology company (BeijingWeiShenQingHong Technology CO., Ltd), we developed an open access survey system (XD.Phone survey management system) and corresponding program for the smartphone. The survey system was established on the server of the company. We typed all the paper questionnaire questions and answer options into the system and checked it to ensure that the smartphone questionnaire was exactly the same as the pen-and-paper questionnaire. When the questionnaire on the survey system was completed, we installed the software program and downloaded the questionnaire on each smartphone.

During our pilot study in July 2011, we did usability and user acceptance testing of the smartphone questionnaire. We encountered some problems, including instability of the

system that made the program shut off, bugs that caused loss of questions when they were downloaded from the server, and mistakes in the responses which were beyond the right range. Based on these experiences, we improved the system together with the information technology company and we adapted the training course for the interviewers.

Figure MA5 Flowchart of data process of smartphone.

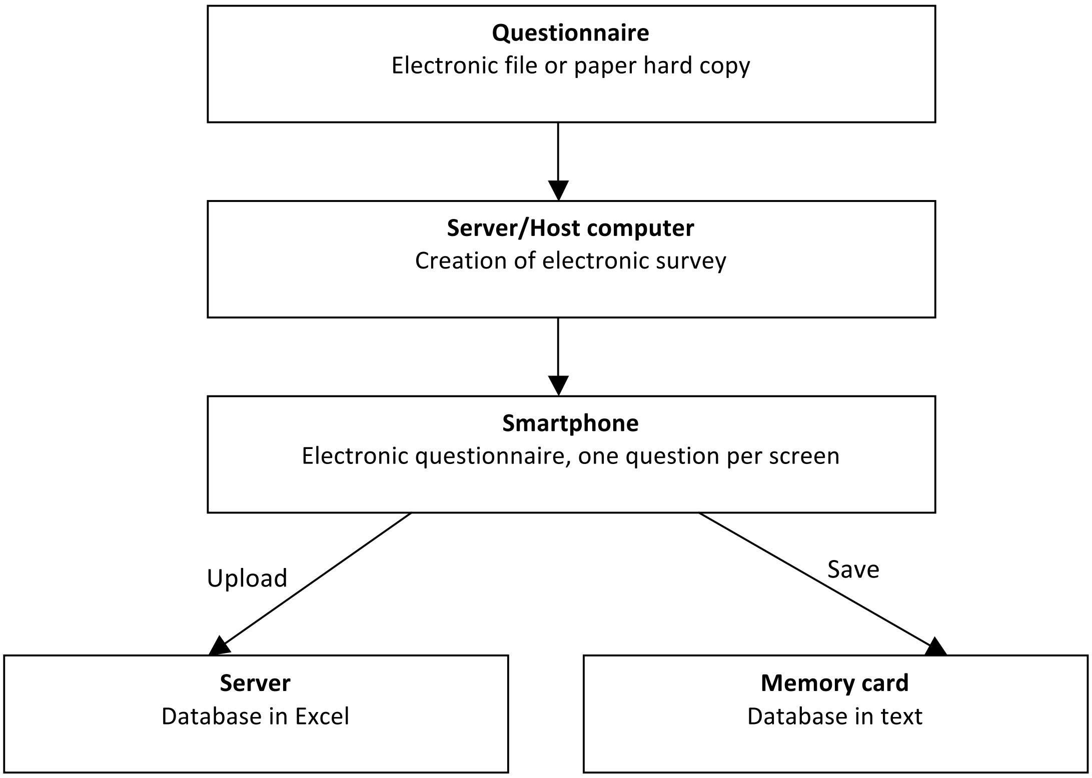

Supplement: Supplementary file 1 [file jmir_v14i5e119_app1.pdf]
