# Supplementary material for: Smartphone Versus Pen-and-Paper Data Collection of Infant Feeding Practices in Rural China
Source: J Med Internet Res. 2012 Sep 18;14(5):e119. doi: 10.2196/jmir.2183 (PMC3510690; doi:10.2196/jmir.2183)
Supplement: Supplementary file 2 [file jmir_v14i5e119_app2.pdf]

More detailed information on the Results section of the paper

Table MA3 Mean time duration of the interviews

		Total; minutes (SD)	Smartphone; minutes (SD)	Pen-and-paper; minutes (SD)	*P*-value[a]
Overall		10.60 (2.49)	10.22(2.17)	10.83(2.94)	.19
Per village	1	11.86 (3.20)	10.78 (2.37)	13.78 (3.70)	.02[b]
	2	10.60 (2.49)	10.61 (2.08)	10.59 (2.83)	.98
	3	9.60 (2.00)	9.23 (1.97)	9.23 (2.06)	.41
	4	9.81 (2.00)	9.50 (1.97)	10.09 (2.07)	.51
Per interview pair	1	12.33 (2.64)	11.82 (2.58)	12.77 (2.71)	.39
	2	9.83 (2.00)	9.70 (1.53)	9.93 (2.34)	.79
	3	10.79 (2.43)	10.33 (2.27)	11.25 (2.60)	.37
	4	10.72 (3.03)	10.81 (1.64)	10.64 (4.23)	.89
	5	8.94 (1.39)	8.68 (1.58)	9.30 (1.06)	.29

[a]Two tailed t-test
[b] $P<.05$

Table MA4 Detailed general information of four surveys in Zhao County

Survey number	Year	Type	No. of variables	No. of completed questionnaires	No. of field work days	No. of interviewees	No. of interviewers	No. of supervisors
1	2011	Smartphone	210	120	1	60	10	2
2	2011	Pen-and-paper	208	120	1	60	10	2
3	2011	Smartphone	1149	1600	12	1600	30	5
4	2010	Pen-and-paper	668	1200	12	1200	25	5
